# Drug Delivery From Polymer-Based Nanopharmaceuticals—An Experimental Study Complemented by Simulations of Selected Diffusion Processes

**DOI:** 10.3389/fbioe.2019.00037

**Published:** 2019-03-08

**Authors:** Innocent J. Macha, Besim Ben-Nissan, Elena N. Vilchevskaya, Anna S. Morozova, Bilen Emek Abali, Wolfgang H. Müller, W. Rickert

**Affiliations:** ^1^Department of Mechanical and Industrial Engineering, University of Dar es Salaam, Dar es Salaam, Tanzania; ^2^Institute of Mechanics, Faculty V of Mechanical Engineering and Transport Systems, Berlin University of Technology, LKM, Berlin, Germany; ^3^Faculty of Science, School of Life Sciences, University of Technology Sydney, Sydney, NSW, Australia; ^4^Applied Research Laboratory, Department of Theoretical Mechanics, Institute for Problems in Mechanical Engineering of the Russian Academy of Sciences and Peter the Great St. Petersburg Polytechnic University, St. Petersburg, Russia

**Keywords:** gentamicin, biphosphonate, polylacetic acid, diffusion coefficient, modeling

## Abstract

The success of medical therapy depends on the correct amount and the appropriate delivery of the required drugs for treatment. By using biodegradable polymers a drug delivery over a time span of weeks or even months is made possible. This opens up a variety of strategies for better medication. The drug is embedded in a biodegradable polymer (the “carrier”) and injected in a particular position of the human body. As a consequence of the interplay between the diffusion process and the degrading polymer the drug is released in a controlled manner. In this work we study the controlled release of medication experimentally by measuring the delivered amount of drug within a cylindrical shell over a long time interval into the body fluid. Moreover, a simple continuum model of the Fickean type is initially proposed and solved in closed-form. It is used for simulating some of the observed release processes for this type of carrier and takes the geometry of the drug container explicitly into account. By comparing the measurement data and the model predictions diffusion coefficients are obtained. It turns out that within this simple model the coefficients change over time. This contradicts the idea that diffusion coefficients are constants independent of the considered geometry. The model is therefore extended by taking an additional absorption term into account leading to a concentration dependent diffusion coefficient. This could now be used for further predictions of drug release in carriers of different shape. For a better understanding of the complex diffusion and degradation phenomena the underlying physics is discussed in detail and even more sophisticated models involving different degradation and mass transport phenomena are proposed for future work and study.

## 1. Introduction

Over the years different drug carriers have been developed and tested for drug delivery and targeting applications. In terms of materials, polymers are the ones mostly used, perhaps due to their simple forming properties in combination with easily tunable properties. In drug release the release phenomenon varies in complexity depending on the design and types of materials involved. For polymeric materials the mechanisms of drug release are normally directly linked to drug diffusion, dissolution, and degradation of the carrier matrix. However, other factors, such as interactions of the material and the drug, can also influence the release kinetics. In addition to physicochemical and morphological properties, the drug location within the matrix, and the drug solubility are key parameters governing the release kinetics and, therefore, the efficiency and efficacy of the treatment. It has been suggested that degradable materials could provide a steady and tunable release kinetics for different therapeutic applications. Furthermore, it was postulated that the use of combinatory materials for the design of drug release systems has the potential for improving drug bioavailability together with predictable release kinetics. Many efforts have been directed toward the development of biodegradable composite materials for drug delivery and targeted controlled release in terms of reproducible and predictable release kinetics in order to meet the therapeutic demands (Ginebra et al., 2006; Habibe et al., 2009; Zhang et al., 2017; Li J. et al., 2018a,b; Li S. et al., 2018; Zhang W. et al., 2018a,b; Zhang Y. et al., 2018). Previous studies showed that bulk eroding polymeric materials show a drug release pattern ranging from one stage (Schmidt et al., 1995; Krasko et al., 2007; Billon-Chabaud et al., 2010; Morawska-Chochółet al., 2014), three stages (Schnieders et al., 2006; Xu and Czernuszka, 2008; Gosau and Müller, 2010) to four stages (Shen et al., 2002; Takenaga et al., 2002). Further studies indicate that the degradation mechanism greatly influences the controlled release of the drug.

This study focuses on the release of gentamicin and clodronate disodium bisphosphonate embedded or not in hydroxyapatite within in a polylactic acid matrix. Similar systems have already been used and are until now in the focus of medical interest (e.g., for the case of gentamicin in Schmidt et al., 1995; Kanellakopoulou and Giamarellos-Bourboulis, 2000; Friess and Schlapp, 2002; Wang et al., 2004; Naraharisetti et al., 2005; Schnieders et al., 2006; Krasko et al., 2007; Xu and Czernuszka, 2008; TorresGiner et al., 2012; Morawska-Chochółet al., 2014; Shim et al., 2015; Dorati et al., 2016; and for bisphosphonate in Billon-Chabaud et al., 2010; Su et al., 2011; Miladi et al., 2013; Papathanasiou and Demadis, 2015; Aderibigbe et al., 2016; Macha et al., 2017a,b; Sovány et al., 2017). It is fair to say that these are experimentally oriented papers written in the spirit of chemistry, biology, and medicine. They are not theoretically oriented. In fact, experimentally obtained release curves are often just presented and sometimes quantified in terms of a very simple diffusion *ansatz* based on the solution of the Fickean diffusion equation for a point source (e.g., Brazel and Peppas, 2000; TorresGiner et al., 2012), which does not truly take the effect of the carrier geometry into account.

However, a considerable effort has also been made for modeling the degradation-drug release behavior in these and other drug carrying systems. One of the objectives is to enable and to accompany a fast and rational design of soluble drug release devices (see e.g., Lee, 1980; Brazel and Peppas, 2000; Siepmann et al., 2002; Raman et al., 2005; Arifin et al., 2006; Lao et al., 2008, 2011; Rothstein et al., 2008, 2009, 2012; Siepmann and Siepmann, 2008; Dash et al., 2010; Fredenberg et al., 2011; Kaunisto et al., 2011, 2013; Siepmann and Peppas, 2012; Hines and Kaplan, 2013). In order to improve the descriptions of drug release some of the researchers included equations governing pore formation and growth. Moreover, different geometries have been investigated for drug release modeling. For example, thin film, spherical, cubical, and cylindrical symmetries are frequently investigated (Lao et al., 2008, 2011; Siepmann and Siepmann, 2008).

If the symmetry of the considered carrier proves to be high then it becomes possible to reduce the simulation problem to the solution of a transient partial differential equation with one spatial dimension (e.g., Siepmann and Siepmann, 2008). The corresponding numerical evaluation is relatively easy and nowadays feasible with conventional computational power. In the case of more complex situations, such as pore formation and growth in the matrix, transport of the drug through an embedding containment as a supply to the matrix (see below), or for truly multi-dimensional geometries the controlled release is more difficult to capture. Concretely this fact is a still a weakness in modeling the drug release and we will discuss as well as suggest further approaches for addressing such phenomena. It is also a fair statement that the community of continuum theoreticians and constitutive theory modelers are not fully aware of the need for capturing the drug release behavior in degrading matrices mathematically.

This paper is a first preliminary attempt to create this awareness. Initially experimental findings will be presented and then correlated with an essentially analytical diffusion model, which explicitly accounts for the underlying drug carrier geometry and, hence, becomes more than just a curve fit. In fact the predicted diffusion coefficients can be considered as geometry independent and useful when assessing the release times from other drug carriers of different geometry.

## 2. Phenomenological Description of Drug Release

In this section the investigated drugs, their containment in hydrolyzable, polymer-based matrices (the carriers), the involved dissolution into a body-like fluid, and the corresponding measurement of the drug concentration in that fluid as a function of time will be described. It will be shown that various stages must be distinguished and a (verbal) explanation for their occurrence will be given.

### 2.1. Drugs, Drug Containing Materials, and the Body Fluid Surrogate

In the experiments the dissolution behavior of two different drugs was investigated, namely of GentaMicin (GM) and of clodronate disodium BisPhosphonate (BP). Some information about their chemical properties can be found in Table 1. In context with the follow-up comparison of diffusion coefficients it is already now important to note that the BP has a smaller molecular mass than the GM molecule. This will have an impact on its migration capabilities through the matrix or, in phenomenological terms, be reflected in the value of the diffusion constant. This important fact has been emphasized before (e.g., in Makadia and Siegel, 2011).

**Table 1:** Chemical composition of drugs and drug carrier matrix.

**Name**	**Chemical formula**	**Molecular structure**	**Molecular weight in g/mol**	**Other properties**
Gentamicin (drug 1)	C_21_H_43_N_5_O_7_	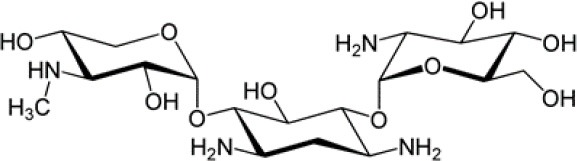	477.6	Solubility in H_2_O: 50 mg/ml
Clodronate disodium bisphophonate (drug 2)	CH_2_Cl_2_Na_2_O_6_P_2_	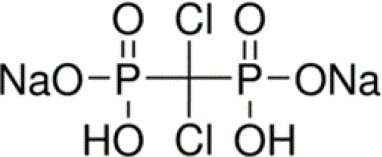	287.85	Soluble in H_2_O
Polylactic acid (matrix)	(C_3_H_4_O_2_)_*n*_	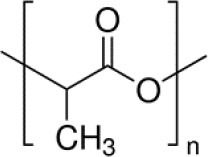	74 250	Crystallinity ≈35%

Two different release media mimicking the body fluid were used for dissolution and diffusion, i.e., phosphate buffered saline solution for the GM and a tris-HCl buffer solution for the BP. The reason for this choice was the procedure applied when measuring the concentration of the released drug, which was sensitive to phosphorus (see the description in section 2.2). Both solutions had a pH of 7.4 and were kept at a “body temperature” of 37 ± 0.1°C. The drugs were stored in a nanoporous matrix made of PolyLactic Acid (PLA), either directly or first embedded in HydroxylApatite (HAp). More specifically coralline HAp was used, and the interested reader can find more information on this topic in corresponding publications by the authors (Ben-Nissan, 2003; Choi et al., 2014). The PLA matrix slowly disintegrates and degrades in the solutions, thereby opening more and more pore space for the drugs to release and also leave their containment eventually.

Choosing a matrix without or with drug embedding and two solutions led to four different experimental scenarios with corresponding concentration measurements, namely GentaMicin contained in PolyLactic Acid (PLA GM), GentaMicin loaded in HydroxylApatite and then contained in PolyLactic Acid (PLA HAp GM), BisPhosphonate contained in PolyLactic Acid (PLA BP), and, finally, BisPhosphonate (BP) loaded in HydroxylApatite (HAp) and then contained in PolyLactic Acid (PLA HAp BP). We proceed to explain the details of the measurements.

### 2.2. Experimental Procedure for Measuring the Concentration of Released Drug

**Figure 1:**
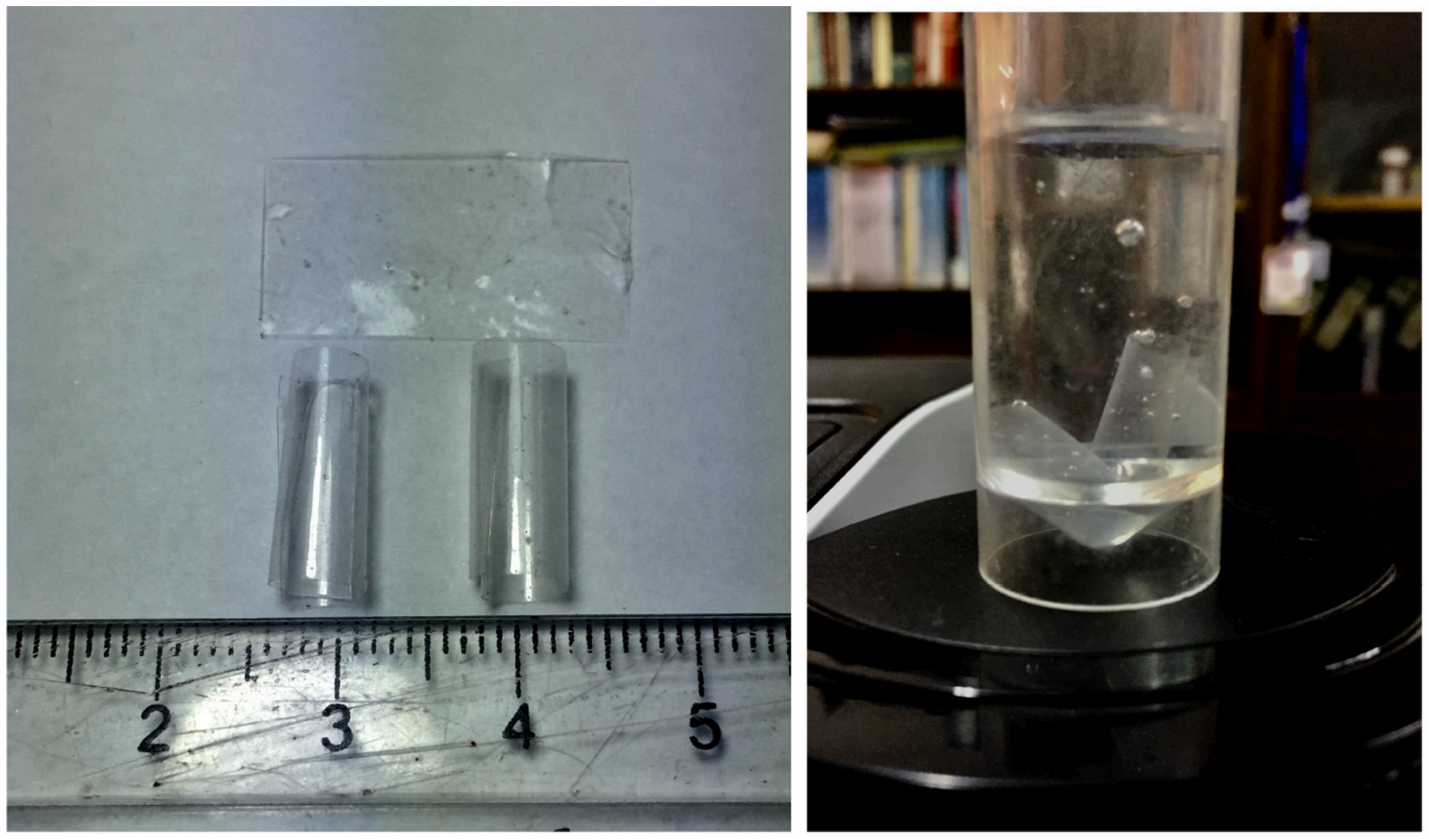
**Left**: Film matrix specimens before and after curling; **Right**: specimens in a tube.

Drug loading to hydrothermally converted coralline HAp was conducted in a vacuum controlled rotavapor with the appropriate amount of either GM or BP mixed with HAp particles to give 10%w/w drug loading. The solution casting method was used during the development of the polymer film composites (either just enriched with the drug or with HAp loaded with drugs) where the PLA was first dissolved in chloroform under room temperature. Then it was mixed with drugs or HAp particles under a magnetic stirrer. After that it was sonicated for 10 min and casted on a petri dish. The solvent in the casted samples was allowed to evaporate under vacuum for 48 h. Finally a thin polylactic acid composite film resulted, which was cut into 2 cm pieces, the thickness of which was around 0.2 mm, *cf*., Figure 1, left.

**Figure 2:**
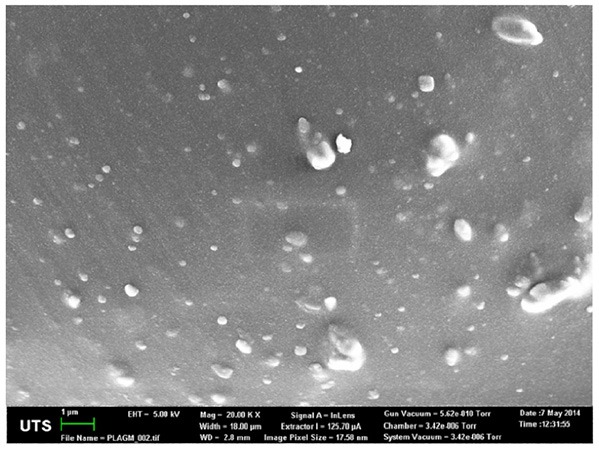
SEM of the drug containment.

Our assumption is that the drug is homogeneously distributed within the containment. This is confirmed by the Scanning Electron Micrograph (SEM) shown in Figure 2. The gray color shows the PLA matrix, and the blisters consist of GM embedded within the matrix. The distribution of the drug is fairly random throughout the matrix, such that the probability of finding drug at any point in the polymer matrix could be constant at all positions within the matrix on the homogenized continuum level of our future models.

The drug release was conducted in a buffer solution at a volume large enough to provide complete dissolution of the drug loaded in the samples. The concentration of drug in buffer solutions was measured by using a UV-vis spectrophotometer (Agilent Technologies, Australia) for GM. The quantification of released BP was determined by using 31P-NMR (Agilent Technologies, Australia).

The raw data of the current (average) concentrations of the drug, $\tilde{c}\left(t\right)$, were originally obtained in units of mg/ml, because by definition the average concentration is given by

(1) $$\begin{array}{l} \tilde{c}(t)=\frac{m(t)}{V_{\text{s}}}, \end{array}$$

where *m*(*t*) is the mass of drug at time *t* within the release medium of volume, *V*_*s*_. Note that the initial amount of drug in the matrices was m_d_ = 7.5mg and the volume of the solution was *V*_*s*_ = 15 ml. Hence the maximum to-be-expected drug release concentration could only be *c*_max_ = 0.5mg/ml. This information could then be used to compute the fraction of drug released at time *t* from the ratio

(2) $$\begin{array}{l} F(t)=\frac{\tilde{c}(t)}{c_{\max}},\;0\leq F(t)\leq 1 \end{array}$$

in dimensionless units.

More specifically, the release medium in liquid form of 15 ml was filled into Falcon™ conical tubes of 17 mm diameter and 120 mm length (see Figure 1, right). Note that the thin film matrix specimens curled up to form cylindrical tubes with a diameter in-between 0.5 and 0.65 cm. Hence they could easily be accommodated in the Falcon™ tube after an initial slight bending. This was possible without breakage because the films were quite flexible. At each time, *t*, 3 × 3 data points for $\tilde{c}$ were taken: The contents of three tubes was examined at every sampling time, such that they all were under the same conditions. As indicated above the concentration values were determined by measuring the absorbance in a UV-vis spectrophotometer. A standard curve was used in this context. It was constructed by using known concentration and measuring the corresponding absorbance. Then the absorbance was plotted against drug concentration resulting in a straight line curve. Spectrophotometer cuvettes were used to store three samples from each tube for the absorbance measurement. As required by the spectrophotometer the cuvettes contained exactly 2 ml. It is fair to say that all nine readings per sampling time were very similar, which explains the small error bars. The contents of each tube was discarded after measurements. Each sampling time had its own three tubes subjected to similar experimental conditions according to the SINK conditions for drug release (SINK, 2009), which require to maintain the release medium and do not allow refills since this would affect the release kinetics.

### 2.3. Graphical Representation of Raw Data

**Figure 3:**
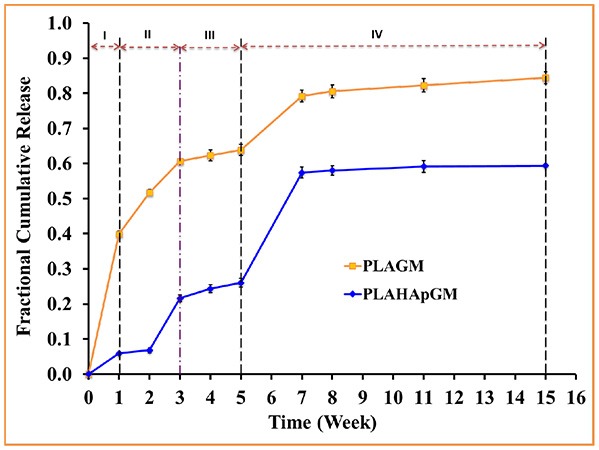
Fractional cumulative release of GM from PLA thin film composite in PBS solution (pH 7.4, 37°C and 100 rpm) for fifteen weeks. Error bars refer to mean standard deviation of triplicate experimental data.

**Figure 4:**
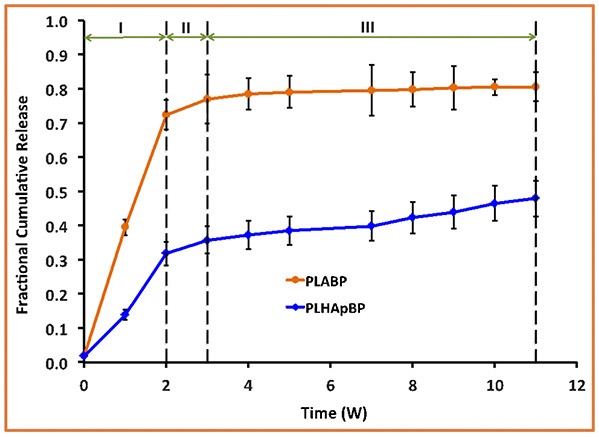
Fractional cumulative release of BP from PLA thin film composite in TrisHCl buffer solution, pH 7.4, at 37°C and 100 rpm for 11 weeks. Error bars refer to mean standard deviation of replicate experimental data.

The experimentally determined fraction of cumulative release, *F*(*t*), for both drugs, GM and BP, are presented in Figures 3, 4, respectively. In each case the influence of a containment for the drug has also been recorded: pure PLA filled with drug *vs*. PLA containing HAp with drug stored within. The continuous lines are (linear) interpolations between the measurement data (squares). Error bars are indicated as well. In order to rule out any measurement related discrepancies, two trial measurements without drug were performed. From these it was concluded that there was no GM present in the (initial) solution, as expected.

**Figure 5:**
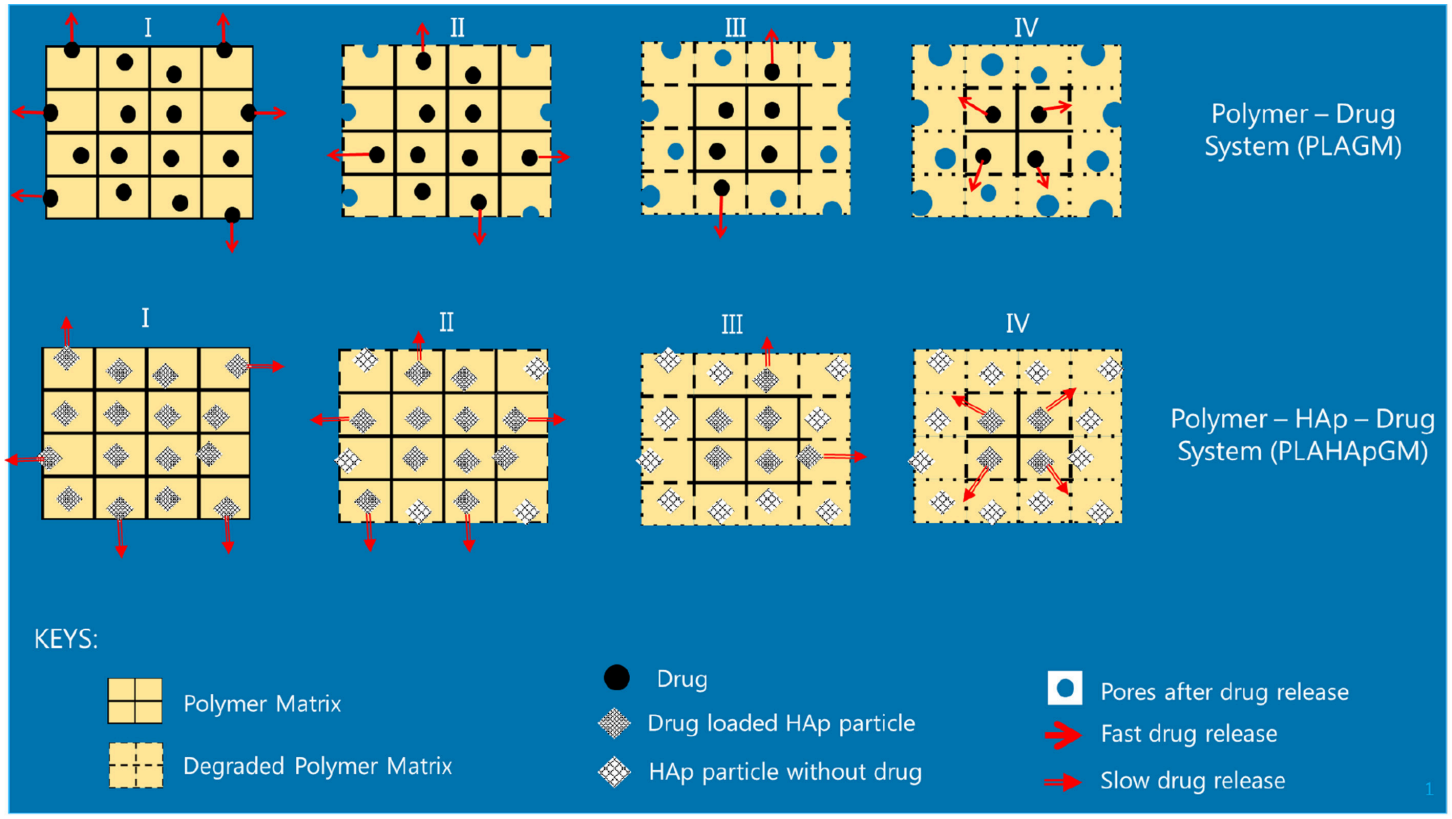
Illustration of release stages for GM without and with HAp.

The different slopes in the plots indicate that it becomes necessary to distinguish different stages of drug release due to different physical phenomena. We will now attempt to give reasons for the observed behavior based on the schematics shown in Figure 5.

Evidently, the experiments with GM easily allow to distinguish several different stages of drug release. In fact one can note four different ones, identifiable by changes in slope or jumps in slope in the release curve. In order to provide some physical justification for this phenomenological observation, we argue as follows:

- Stage I (week 1): In the case of the PLA GM measurements, we observe initially a burst. This accelerated release is due to drug particles situated on the outer surface of the matrix migrating into the release medium. The illustration in the first top inset of Figure 5 visualizes this phenomenon. For PLA HAp GM the “burst” is less significant than for PLA GM. We explain this fact as “configuration related,” this is to say related to the confinement of the drug with the HAp. The drug—even if close to the surface of the matrix—still has to tunnel through the HAp and be supplied to the PLA-matrix, see the first bottom inset in Figure 5.
- Stage II (weeks 1–3): PLA GM continues to show a significant release of drug, but the speed of release, i.e., the slope to the *F*(*t*) curve does not show a jump. The polymer matrix will start to degrade close to the surface and the corresponding drug as well as some drug from further inside will be released, see the second top inset in Figure 5. This behavior is analogous for PLA HAp GM but not as pronounced, because of the HAp, which must be overcome first to supply the drug, which can then diffuse through the matrix, second bottom inset in Figure 5. Also between week 2 and 3 a sudden burst is observed. Some additional “production” of GM must be the reason, maybe due to deterioration of the HAp embedding, at least close to the surface.
- Stage III (weeks 3–5): Both PLA GM and PLA HAp GM show a stagnation of drug release. One might think that the process reaches an equilibrium. However, Stage 4 (see below) causes us to develop another hypothesis. This lag phase can be explained by the fact that the PLA matrix keeps degrading; but all of the drug close to the surface has already been released, see the third top and bottom insets in Figure 5.
- Stage IV (weeks 5–15): In both cases the polymer matrix finally starts to show strong degrees of deterioration, pores are forming through which the GM or the supply from the HAp GM can more easily diffuse, see fourth top and bottom insets in Figure 5. The experiments were stopped after week 15. Note that the release process in the case of PLA HAp GM is much more pronounced as in the case of PLA GM. This is due to the breaking of the HAp skeleton in addition to a strongly deteriorating PLA matrix. This fact will affect our future modeling.

In case of the BP experiments presented in Figure 4, no trial measurements without drug were performed. The tests seem to indicate a more or less continuous release of drug. In contrast to GM at most three stages can be observed. The lag phase is missing or not as pronounced, which may be due to the fact of the different diffusion properties of the smaller BP molecules. More specifically we note:

- Stage I (weeks 1 and 2): A strong burst of drug release is visible in the case of PLA BP. The burst is much more pronounced than for the case of PLA HAp BP, since in the latter configuration it is necessary to overcome the additional HAp barrier first and to supply drug to the polymer matrix. It also shows that the drug release for PLA GM and PLA HAp GM is less than for PLA BP and PLA HAp BP. Obviously the diffusion in the two latter cases is easier, maybe due to the smaller size of the BP drug molecules.
- Stage II (week 2–3): The drug release slows down in both cases, this process can be related to an equilibrium condition. At the same time, the formation of voids due to the degradation of the matrix is on its way.
- Stage III (week 3–11): In both cases the formation of voids and degradation of the matrix is sluggishly continuing in the case of PLA BP and slightly faster for PLA HAp BP. No strong jumps in the release speed were observed in this and all other stages. This causes us to believe that the corresponding solution fluid might be less aggressive.

In summary we may say that the cases of PLA GM and PLA BP should be describable by standard diffusion equations, whereas in the case of PLA HAp GM and PLA HAp BP a more complex simulation seems to be required. Here the standard diffusion equation should be equipped by a supply term mimicking the provision of drug from the HAp containment to the PLA matrix.

## 3. Modeling of the Diffusion Process

In this paper the emphasis will be on modeling the release processes in context with drug exclusively stored in the PLA matrix through which it diffuses, either slowly as long the polymer stays intact, or more quickly when the polymer gradually deteriorates and gives “more way” to the diffusing drug molecules. More precisely, the results shown in the orange curves of Figures 3, 4 will be quantified in terms of (time-dependent) diffusion constants. The principles of the modeling will be explained in the next subsection and then gradually be made more concrete.

A quantification of the blue curves in both pictures is more difficult, because the underlying physics should be observed in the modeling. Here the drug has to diffuse first through the HAp to enter the PLA matrix. In other words, the drug is supplied to the matrix and after that it diffuses through the PLA into the solution. The supply will be greater when the HAp starts to deteriorate, but it is still a supply, which will be part of a modified diffusion equation, but not in terms of an adjusted diffusion coefficient of drug diffusing through the polymer matrix. Hence this type of modeling will result in diffusion constants and in parameters characteristic of the supply, i.e., the release of drug into the polymer matrix. For the supply term in the diffusion equation an adequate constitutive equation must be stated. The idea is to base this relation on a micro-model for the drug diffusion through an HAp shell. However, for conciseness of this paper the corresponding quantification is left to future research.

### 3.1. Initial Remarks on the Principles of the Modeling Procedure

Some remarks from the viewpoint of continuum physics are now in order. Typically the concentration, *c*, is a field variable: *c*(***x***, *t*). In other words, unlike the measured data, $\tilde{c}\left(t\right)$, it depends on both time, *t*, and spacepoint, ***x***. The experimentally determined concentration was no true field. It was uniform within the solution, because the test was conducted in a water bath temperature controlled shaker at 100 rpm.

In our simulations we will study the diffusion within a thin film matrix. The film is curled up in form of a cylindrical tube and fully immersed in the solution. For this geometry we will solve a transient diffusion equation of the form

(3) $$\begin{array}{l} \frac{\partial c}{\partial t}=-\nabla \cdot J-\dot{d}, \end{array}$$

where ***J***(***x***, *t*) is the diffusion flux for which we use Fick's first law,

(4) $$\begin{array}{l} J=-D_{\text{m}}\nabla c \end{array}$$

with the unknown diffusion coefficient of the drug in the matrix, *D*_m_, which is a true constant for an isothermal process. The mass of the drug is conserved. Hence usually there is no drain term $\dot{d}$. However, we shall see that at later stages of the release process a constant diffusion coefficient would lead to predictions for the concentrations of drug in the solution higher than observed. For this reason we assume that drug particles are absorbed in the matrix, follow (Nestle and Kimmich, 1996) and write:

(5) $$\begin{array}{l} \dot{d}=\frac{\partial c_{\text{b}}}{\partial t}, \end{array}$$

where *c*_*b*_ is the concentration of the bound molecules, a.k.a. the sorption isotherm. Based on fits to experimental data many expressions have been proposed for sorption isotherms linking them to the concentration *c*. We follow the proposition by Temkin (see the list and reference in Table 1 of Nestle and Kimmich, 1996),

(6) $$\begin{array}{l} c_{\text{b}}=A\ln(Bc), \end{array}$$

*A* and *B* being (constant) material parameters. It follows that:

(7) $$\begin{array}{l} \frac{\partial c}{\partial t}=D_{\text{eff}}\Delta c,\;D_{\text{eff}}=\frac{c}{c+A}D_{\text{m}}. \end{array}$$

This is one of the simplest form of an effective diffusion coefficient, *D*_eff_, which depends on the concentration itself. It contains only one additional parameter, *A*, besides the diffusion coefficient.

It should be mentioned that including a drain term in the diffusion equation is not the only way to account for the fact that the outflux of drugs stagnates at higher concentration levels in the solution. Indeed, it is possible to put $\dot{d}=0$ and to base the model on boundary conditions of the Robin type:

(8) $$\begin{array}{l} \frac{\partial \tilde{c}(t)}{\partial t}=-\alpha \left(c^{*}-\tilde{c}(t)\right)\nabla c\cdot n, \end{array}$$

where ***n*** is the outward normal to the boundary surface. Moreover, c^*^ is the observed saturation level of the solution concentration [to be read off from the experimental *F*(*t*) plot], $\tilde{c}\left(t\right)$, and α is the only remaining unknown material parameter characteristic of the permeability of the matrix wall, which is at the drug concentration level *c*. In engineering is also known as the mass transfer coefficient.

The diffusion flux can be used to obtain the mass outflux of drug, *ṁ*(*t*), across the surfaces of the cylindrical tube, ∂*V*_cyl_,

(9) $$\begin{array}{l} \dot{m}(t)=\oint_{\partial V_{\text{cyl}}}J(x,t)\cdot n\text{ d}A, \end{array}$$

where d*A* is the surface element.

Hence the total mass of drug released into the solution follows by integration in time from which the drug release can be calculated:

(10) $$\begin{array}{l} m(t)=\int_{\tilde{t}=0}^{\tilde{t}=t}\dot{m}(t)\text{ d}t\;\Rightarrow \;F(t)=\frac{m(t)}{V_{\text{s}}c_{\text{max}}}. \end{array}$$

The unknown diffusion constant can now be determined such that the predicted drug release agrees with the actually observed average release fraction *F*(*t*), which is known from the experiments. The necessary input data will be discussed shortly.

In the next subsections we present solutions to these various initial boundary value problems. We will start with a closed form solution for the concentration field *c*(***x***, *t*) at a point ***x*** within the matrix tube. This expression contains the unknown diffusion constant of the drug in the matrix, *D*_m_, and takes the cylindrical geometry explicitly into account. The advantage of this solution is that everything can be evaluated analytically without taking resource to advanced numerical methods.

### 3.2. Closed for Solution of a Simplified One-Dimensional Diffusion Problem

It was mentioned above that the drug is released from a hollow cylindrical tube, which formed by curling of a square thin film (the “matrix”) of height *h* = 20 mm and thickness *d* = 0.2 mm into a surrounding fluid (the “solution”) of volume *V*_s_ = 15 ml. Strictly speaking, this is a axisymmetric yet three-dimensional problem, because of the finite height of the cylindrical tube, which can only be treated numerically. However, even with the appropriate tool, e.g., a finite element (FE) code, finite volume (FV) or finite difference methods, it will result in a model with a very high amount of degrees of freedom, in particular because of the extremely thin cylinder wall, which needs to be discretized very finely. Surely, such an approach is prohibitive if the objective is a timely check of how a change of matrix geometry (for example) would influence the release time. Hence, it is beneficial to have a simplified simulation tool at one's disposal, which can be exploited without too much computational effort—in other words, a model that allows for a more or less analytical solution.

In this spirit we argue that the diffusion process from the cylindrical tube into the solution takes place only in radial direction and the transport in axial direction of the tube walls to the solution can be ignored. This seems reasonable, because the cylinder walls are so thin when compared to height of the cylinder axis.

The ordinary Fickean diffusion equation for the concentration field *c* follows from Equation (7) by putting *k* = 0. Then for a purely radial dependence of the concentration the cylindrical Laplace operator reads when applied to *c*(*r, t*):

(11) $$\begin{array}{l} \Delta c=\frac{1}{r}\frac{\partial }{\partial r}\left(r\frac{\partial }{\partial r}\right)=\frac{\partial^{2}c}{\partial r^{2}}+\frac{1}{r}\frac{\partial c}{\partial r}. \end{array}$$

We will solve this equation only within the wall of a now infinitely long cylindrical tube, i.e., within the region *a* = *r*_i_ ≤ *r* ≤ *b* = *r*_i_ + *d*, where the inner radius is given by:

(12) $$\begin{array}{l} r_{\text{i}}=\frac{h}{2\pi }\approx 3.2\text{ mm.} \end{array}$$

The initial concentration within the cylinder walls is a constant and given by:

(13) $$\begin{array}{l} c_{0}=\frac{{\text{m}}_{\text{d}}}{\pi h\;\left(2r_{\text{i}}d+d^{2}\right)}\approx 90.9\frac{\text{mg}}{\text{ml}}. \end{array}$$

**Figure 6:**
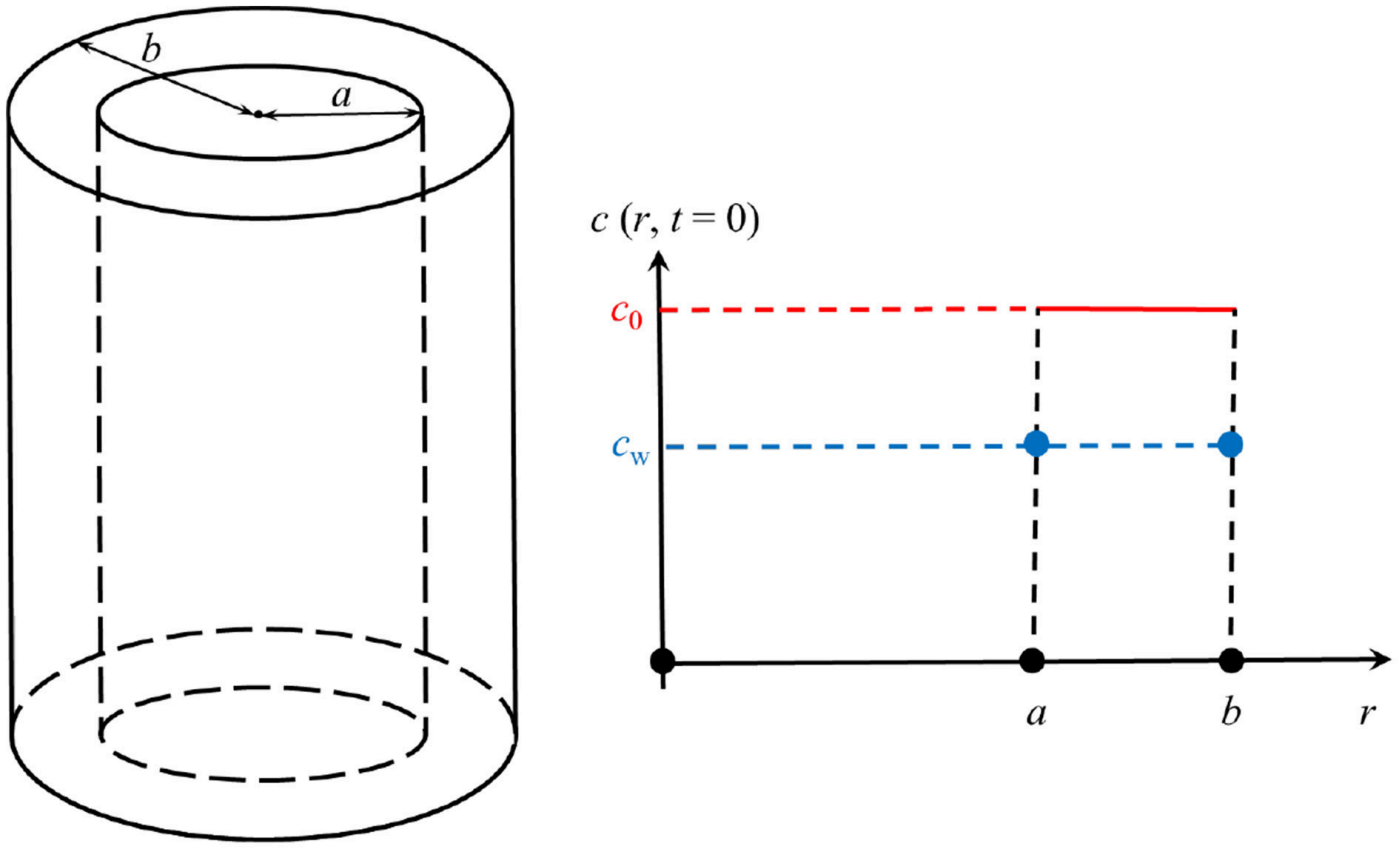
Illustration of the geometry.

The situation is illustrated in Figure 6. This in combination with

(14) $$\begin{array}{l} \frac{\partial c}{\partial t}=D_{\text{m}}\left(\frac{\partial^{2}c}{\partial r^{2}}+\frac{1}{r}\frac{\partial c}{\partial r}\right),\;a\leq r\leq b, \end{array}$$

where *D*_m_ is the (unknown) diffusion coefficient in the matrix, forms the skeleton of our initial-boundary-value-problem. The question remains, which boundary conditions to employ. To this end it is argued as follows. First, assume that the diffusion within the liquid is much faster than within the solid matrix, which seems reasonable. Then conclude that the flux of mass leaving the solid at the boundaries is immediately distributed evenly within the solution, inside and outside of the cylinder, because in the real experiment these regions are connected anyway. Following Equation (4) one may say that, in general, the flux in a purely radial cylindrically symmetric problem is given by

(15) $$\begin{array}{l} J=-D_{\text{m}}\frac{\partial c}{\partial r}e_{r}. \end{array}$$

Hence the total output of mass per unit time across the boundaries at *a* and *b* reads

(16) $$\begin{array}{l} \dot{m}(t)=h2\pi D_{\text{m}}\left({\frac{\partial c}{\partial r}|}_{r=a}a-{\frac{\partial c}{\partial r}|}_{r=b}b\right). \end{array}$$

This will lead to a rise of drug concentration in the solution of d*c* = ^*ṁ*d*t*^/_*V*_s__ and we may conclude that the concentration at the boundaries is a function of time and given by

(17) $$\begin{array}{l} c(t)=\frac{1}{V_{\text{s}}}\int_{\tilde{t}=0}^{\tilde{t}=t}\dot{m}(t)\text{ d}t. \end{array}$$

In fact, if we divide this expression by *c*_max_ we obtain the drug release function *F*(*t*) defined in Equation (2) and numerical values for this function are known from the experiments shown in Figures 3, 4. This concludes detailing the relations shown in Equations (9)–(10) for the case of a cylindrical tube, the axis of which is much longer than the thickness of its walls. Hence all that remains is to find a solution of the diffusion Equation (14) with time-dependent boundary conditions:

(18) $$\begin{array}{l} c(r=a,t)=F(t)c_{\max},\;c(r=b,t)=F(t)c_{\max}. \end{array}$$

To find this solution is unfortunately also a numerical task. However, if the boundary conditions are time-independent and given by the value *c*_w_ (see Figure 6), an analytical solution of the cylinder symmetric diffusion problem was presented in Carslaw and Jaeger (1959, p. 205), section 7.10, based on Bernoulli's method of the separation of variables, here *r* and *t*. Rewritten for our purposes it reads

(19) $$\begin{array}{l} c(r,t)=c_{\text{w}}+\pi \left(c_{0}-c_{\text{w}}\right)\sum_{n=1}^{\infty }\frac{J_{0}\left(\alpha_{n}\right)U_{0}\left(\frac{r}{a}\alpha_{n}\right)}{J_{0}\left(\alpha_{n}\right)+J_{0}\left(k\alpha_{n}\right)}\;\exp\left(-\frac{D_{\text{m}}\alpha_{n}^{2}}{a^{2}}t\right), \end{array}$$

where

(20) $$\begin{array}{l} U_{0}\left(\frac{r}{a}\alpha_{n}\right):=J_{0}\left(\frac{r}{a}\alpha_{n}\right)Y_{0}\left(k\alpha_{n}\right)-J_{0}\left(k\alpha_{n}\right)Y_{0}\left(\frac{r}{a}\alpha_{n}\right),\;k:=\frac{b}{a}, \end{array}$$

and *c*_0_ is the initially constant concentration within the wall of the cylindrical tube. *J*_0_ and *Y*_0_ are Bessel functions of the first and second kind of zeroth order. The coefficients α_*n*_ are roots to the following transcendental equation

(21) $$\begin{array}{l} U_{0}\left(\alpha_{n}\right)=0. \end{array}$$

They must be found numerically. It turns out that for our case of a very thin wall, *k* ≈ 1.063, they are very large. A numerical investigation using Mathematica shows that α_*n*_ ≈ 50 *n*. This means in practice that, because of the exponential, we can already cut off the infinite series after the first term. It also facilitates getting an estimate for the diffusion data, as we shall see shortly. To begin with we use the solution shown in Equation (19) in context with Equation (16) to find the mass rate

(22) $$\begin{array}{l} \dot{m}(t)=4\pi hD_{\text{m}}\left(c_{0}-c_{\text{w}}\right)\sum_{n=1}^{\infty }\frac{J_{0}\left(\alpha_{n}\right)-J_{0}\left(k\alpha_{n}\right)}{J_{0}\left(\alpha_{n}\right)+J_{0}\left(k\alpha_{n}\right)}\;\exp\left(-\frac{D_{\text{m}}\alpha_{n}^{2}}{a^{2}}t\right). \end{array}$$

During the derivation of this formula use was made of the relations

(23) $$\begin{array}{l} {\left(r\frac{\text{d}U_{0}}{\text{d}r}\right)|}_{r=a}=-\frac{2}{\pi }\frac{J_{0}\left(k\alpha_{n}\right)}{J_{0}\left(\alpha_{n}\right)},\;{\left(r\frac{\text{d}U_{0}}{\text{d}r}\right)|}_{r=b}=-\frac{2}{\pi }. \end{array}$$

Since during the derivation of Equation (22) *c*_*w*_ and *D*_m_ were assumed to be constant, the expression can easily be integrated over a time interval *t*_*m*_ ≤ *t* ≤ *t*_*m*+1_:

(24) $$\begin{array}{l} \begin{array}{l} m\left(t_{m+1}\right)-m\left(t_{m}\right)=4\pi ha^{2}\;(c_{0}-c_{\text{w}})\times \\ \sum_{n=1}^{\infty }\frac{J_{0}\left(\alpha_{n}\right)-J_{0}\left(k\alpha_{n}\right)}{\alpha_{n}^{2}\left[J_{0}\left(\alpha_{n}\right)+J_{0}\left(k\alpha_{n}\right)\right]}\left[\exp\left(-\frac{D_{\text{m}}\alpha_{n}^{2}}{a^{2}}t_{m}\right)-\exp\left(-\frac{D_{\text{m}}\alpha_{n}^{2}}{a^{2}}t_{m+1}\right)\right]. \end{array} \end{array}$$

Equation (24) forms the basis for our numerical assessment in the next section.

### 3.3. Evaluation of the Simplified One-Dimensional Model and Comparison With the Literature

Table 2 presents the numerical drug release data for GM in PLA shown in the orange curve of Figure 3. They will now be used to obtain an approximative value for the constant of diffusion, depending upon the considered time interval between time step *m* to time step *m* + 1. Assume that *c*_*w*_ = *c*_*w*_(*t*_*m*_). Then we evaluate (24) in a discretized fashion as follows and find for the increase in drug release in the time interval *t*_*m*_ ≤ *t* ≤ *t*_*m*+1_:

(25) $$\begin{array}{l} \begin{array}{c} F\left(t_{m+1}\right)=F\left(t_{m}\right)+\\ \frac{4\pi ha^{2}}{V_{\text{s}}}\frac{c_{0}-c_{\text{w}}\left(t_{m}\right)}{c_{\text{max}}}\sum_{n=1}^{\infty }\frac{J_{0}\left(\alpha_{n}\right)-J_{0}\left(k\alpha_{n}\right)}{\alpha_{n}^{2}\left[J_{0}\left(\alpha_{n}\right)+J_{0}\left(k\alpha_{n}\right)\right]}\\ \left[\exp\left(-\frac{D_{\text{m}}^{m}\alpha_{n}^{2}}{a^{2}}t_{m}\right)-\exp\left(-\frac{D_{\text{m}}^{m+1}\alpha_{n}^{2}}{a^{2}}t_{m+1}\right)\right], \end{array} \end{array}$$

where $D_{\text{m}}^{m+1}$ is an update to the diffusion coefficient at the time $D_{\text{m}}^{m}$. Recall that the drug release data *F*(*t*_*m*+1_) and *F*(*t*_*m*_) are known from the experiments: Table 2.

**Table 2:** GM in PLA.

**Week m**	**0**	**1**	**2**	**3**	**4**	**5**	**7**	**8**	**9**	**11**	**15**
*F*(*t*_*m*_)	0.0	0.399	0.517	0.607	0.623	0.639	0.792	0.805	0.814	0.823	0.844

We are now in a position to compute an update for the diffusion coefficient:

(26) $$\begin{array}{l} D_{\text{m}}^{m+1}=-\frac{a^{2}}{\alpha_{1}^{2}t_{m+1}}\ln[\exp\left(-\frac{D_{\text{m}}^{m}\alpha_{1}^{2}}{a^{2}}t_{m}\right)-\\ \left[F(t_{m+1})-F(t_{m})\right]\frac{V_{\text{s}}}{4\pi ha^{2}}\frac{c_{\max}}{c_{0}-c_{\text{w}}(t_{m})}\frac{\alpha_{1}^{2}\left[J_{0}\left(\alpha_{1}\right)+J_{0}\left(k\alpha_{1}\right)\right]}{J_{0}\left(\alpha_{1}\right)-J_{0}\left(k\alpha_{1}\right)}]. \end{array}$$

**Figure 7:**
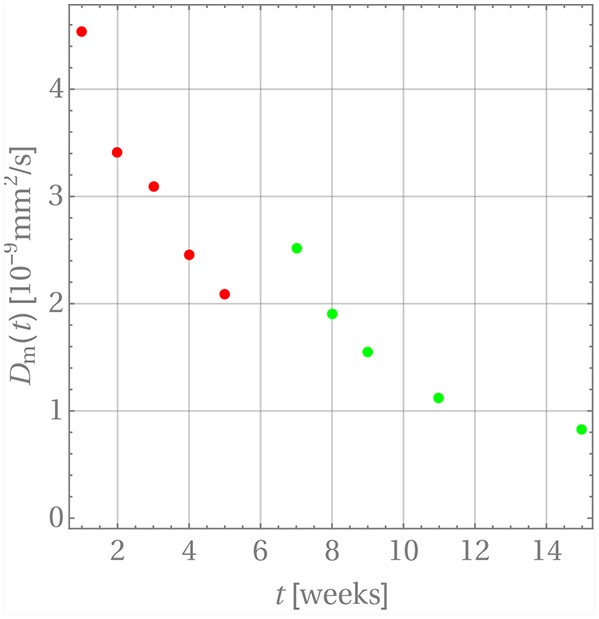
The time dependence of diffusion coefficients for GM in PLA.

For this purpose we have terminated the sum after the first term. This is possible because of the large values of α_*n*_. The starting value for the diffusion coefficients is zero, $D_{\text{m}}^{0}=0$, because there is no diffusion before week 0. The diffusion coefficients resulting for weeks 1 through 5 are indicated by the red dots in Figure 7. A time-dependence accompanied by a steady decrease can be observed, which one may want to interpret intuitively as an inhibition of diffusion, because the difference *c*_0_ − *c*_*w*_(*t*_*m*_) becomes smaller and smaller as time progresses. However, as the discussion below will show, this interpretation should be taken with a grain of salt.

**Figure 8:**
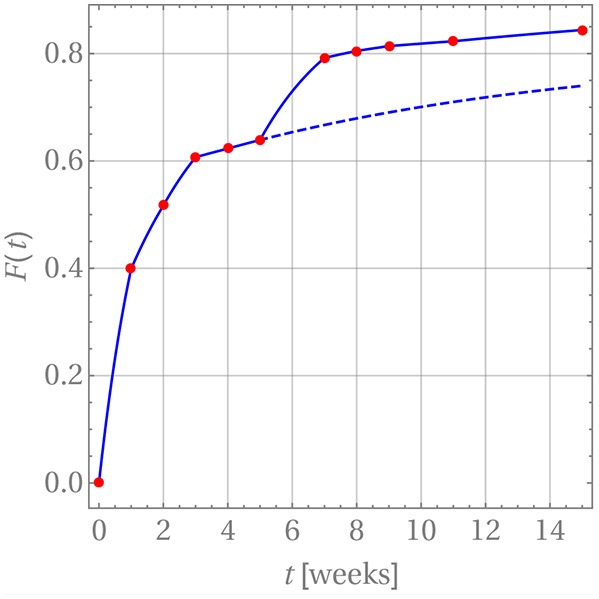
Drug release predicted (blue curve) for GM in PLA in comparison with measurement data (red dots).

Now that the diffusion coefficients are known the progression of drug release can be calculated from the following formula:

(27) $$\begin{array}{l} \begin{array}{l} F_{m+1}(t)=F_{m}\left(t_{m}\right)+\frac{4\pi ha^{2}}{V_{\text{s}}}\frac{c_{0}-c_{\text{w}}\left(t_{m}\right)}{c_{\text{max}}}\sum_{n=1}^{\infty }\frac{J_{0}\left(\alpha_{n}\right)-J_{0}\left(k\alpha_{n}\right)}{\alpha_{n}^{2}\left[J_{0}\left(\alpha_{n}\right)+J_{0}\left(k\alpha_{n}\right)\right]}\times \\ \left[\exp\left(-\frac{D_{\text{m}}^{m}\alpha_{n}^{2}}{a^{2}}t_{m}\right)-\exp\left(-\left[\left(D_{\text{m}}^{m+1}t_{m+1}-D_{\text{m}}^{m}t_{m}\right)\frac{t-t_{m}}{t_{m+1}-t_{m}}+D_{\text{m}}^{m}t_{m}\right]\frac{\alpha_{n}^{2}}{a^{2}}\right)\right], \end{array} \end{array}$$

where *t*_*m*_ ≤ *t* ≤ *t*_*m*+1_. Note that in practice it is not necessary to carry out the summation beyond *n* = 1. In order to introduce a continuous time dependency, the updated drug release is modified, *F*_*m*+1_(*t*_*m*+1_) → *F*_*m*+1_(*t*), and the product $D_{\text{m}}^{m+1}t_{m+1}$ in Equation (25) is replaced by a linear interpolation between the time steps *t*_*m*_ and *t*_*m*+1_. The perfect agreement for weeks 1 through 5 is shown in Figure 8.

After week 5 the last diffusion coefficient was used to predict the drug release if there were no change in the release mechanism. This is indicated by the dashed blue line. Clearly the prediction does not match the actually observed data. Indeed, it has already been noted that after that time the matrix deteriorates strongly. Consequently, a different evaluation strategy for the diffusion is required. In fact, it is still based on Equation (26) with three subtle differences. First, *c*_0_ was replaced with a concentration value based on the amount of drug remaining in the matrix after the release through weeks 1 through 5. Strictly speaking, the concentration profile is no longer constant within *r*_*i*_ ≤ *r* ≤ *r*_*i*_ + *d*. However, *d* is so small that this variation can be neglected and the solution for a constant initial concentration be reused. Second, the diffusion started anew, i.e., a diffusion coefficient *D*_m_ = 0 was used at time *t*_*m*_ = week 5 and, third, *F*(*t*_*m*_ = week 5) = 0.639 was used as starting value for the drug release.

The resulting diffusion coefficients are indicated by the green dots in Figure 7. They also decrease steadily over time and a jump when compared to the last red dot is clearly visible, which is indicative of the change in diffusion mechanism. An accordingly adjusted version of Equation (27) was then used to predict the drug release during weeks 5 through 11, which is shown in Figure 8.

A word of caution is finally in order. Note that there are no physical reasons that the diffusion coefficient decreases during the release process in weeks 1 through 5 and then, after the jump, once more in weeks 5 through 15. The diffusion coefficient should simply remain constant during these two time intervals. Its time-dependent behavior must be attributed to the fact that constant concentrations *c*_*w*_ were used for boundary conditions during the discretization steps, whereas in reality the concentration at the boundary, *c*_*w*_(*t*), is time-dependent and steadily growing, so that the difference *c*_0_ − *c*_*w*_(*t*) becomes smaller over time. Hence the driving force for diffusion, ***J***, actually decreases in magnitude because of a lack in concentration gradient and not because of a time-dependent diffusion constant (see Equation (4)). This is an artifact of our way of simulation and due to the chosen closed-form solution, which does not allow for time-dependent concentrations at the cylinder walls. The only physically meaningful conclusion one may draw from Figure 7 is that there is a boost in drug release at week 5, due to the heavily deteriorated matrix. If one so wishes the diffusion coefficients during weeks 1 through 5 and 5 through 15 can be averaged to obtain ballpark number for describing the diffusion before and after matrix deterioration, ${\bar{D}}_{\text{m}}^{1-5}\approx 3.12\times 10^{-9}{mm}^{2}{/}_{s}$ and ${\bar{D}}_{\text{m}}^{5-15}\approx 1.28\times 10^{-9}{mm}^{2}{/}_{s}$. However, by doing this one loses the aspect of the boost in drug release after week 5.

Performing a comparison between our diffusion coefficients for GM in PLA is not as straightforward as one wishes it to be, mainly for two reasons. First, the diffusion data in the literature are often obtained by fitting release curves with the point source solution of the Fickean diffusion equation (see e.g., Crank, 1979, p. 11), i.e., a $\sqrt{t}$-fit, which is geometry dependent and, second, the matrix chosen for GM is not PLA.

In Dunn et al. (1981, p. 137) values for the product DC_*s*_ are shown, where D is the diffusion coefficient and C_*s*_ is the saturation solubility. For poly (DL-lactide) a value ${\text{DC}}_{s}=6.4\times 10^{-13}\frac{g}{\text{cm s}}$ is presented. If the saturation solubility is assumed to be equal to *c*_0_ a diffusion constant of roughly $7.0\times 10^{-9}\frac{{mm}^{2}}{\text{s}}$ is obtained, which is of the same order as our values.

In Table 1 from Zhang et al. (1995) the release of GM from poly(DL-lactide) full cylinders of various heights, *L*, was investigated and resulted in what the authors called “effective” diffusion coefficients, *D*_eff_, for this particular geometry^1^. By comparing the exponentials showing the temporal evolution for the full cylinder geometry and for the solution used in this paper, one comes to the conclusion that:

(28) $$\begin{array}{l} D_{\text{m}}=\frac{\pi^{2}r_{\text{i}}^{2}}{L^{2}\alpha_{1}^{2}}D_{\text{eff}}. \end{array}$$

This conversion formula was applied to the data leading to the values shown in Table 3. Hence most of the values agree with the findings of this paper.

**Table 3:** Diffusion coefficients *D*_m_ in units of $10^{-9}\frac{\text{m}{\text{m}}^{2}}{s}$ as a function of drug loading converted from data in Zhang et al. (1995).

**L [mm]**	**20 wt%**	**30 wt%**	**40 wt%**	**50 wt%**
2	-	4.8	-	-
4	-	1.7	-	-
5	1.0	1.3	11.1	73.9
7	-	1.5	-	-
10	-	1.0	4.1	41.1
20	-	-	1.6	31.4

**Figure 9:**
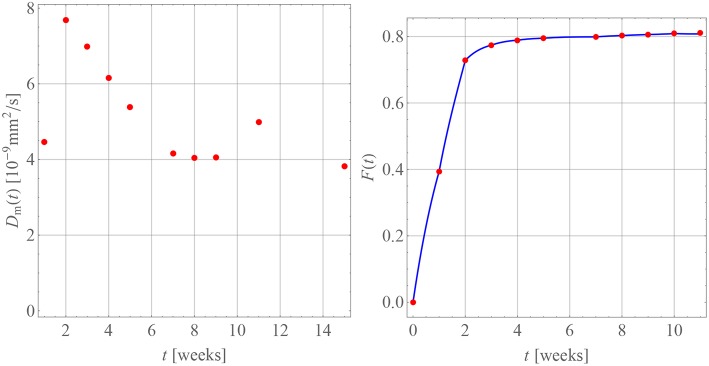
Diffusion coefficients **(Left)** and drug release predicted (**Right**, blue curve) for BP in PLA in comparison with measurement data (red dots).

Table 4 contains the numerical data for the release of BP within PLA as shown in the orange curve of Figure 4. In Figure 9, the results for the corresponding diffusion constants and release predictions are presented, analogously as in the case of GM, Figures 7, 8. The same words of caution apply as in context with Figure 7: The time-dependence of the diffusion constants does not have a physical meaning. Rather it is an artifact of the closed-form solution used, which does not allow for time-dependent boundary conditions. However, we may say that tendentially the diffusion constants (left inset of the figure) are now higher because the drug molecule is smaller. And what is more, there is no abrupt jump of the diffusion coefficients after some weeks. Moreover, the fact that the first and ninth diffusion coefficient in the left inset of Figure 9 show some unsteady behavior should be attributed to the discretized fitting procedure and also not be regarded as a physical effect. In fact note that at high times the slope of the drug release curve becomes quite shallow, which is a problem for the applied discrete evaluation technique. But finally, as in the case of GM, an excellent agreement for the predicted release rates with the experimental measurements (inset on the right) can be observed.

**Table 4:** BP in PLA.

**week m**	**0**	**1**	**2**	**3**	**4**	**5**	**7**	**8**	**9**	**10**	**11**
*F*(*t*_*m*_)	0.0	0.394	0.728	0.774	0.789	0.795	0.799	0.803	0.806	0.809	0.811

A comparison of our diffusion constants for BP in PLA with values from the literature is very difficult, since despite an intensive search such data seems currently not to be available. Billon-Chabaud et al. (2010) present experimental release curves of BP from various carriers but do not evaluate them mathematically. Based on studies of the C14 migration of zoledronate through trabecular bone (Stadelmann et al., 2009) report an estimate for a diffusion coefficient of $9.3\times 10^{-9}\frac{\text{m}{\text{m}}^{2}}{s}$, which despite the different medium is of the same order of magnitude as our data.

### 3.4. Numerical Solutions of the Diffusion Problems

**Figure 10:**
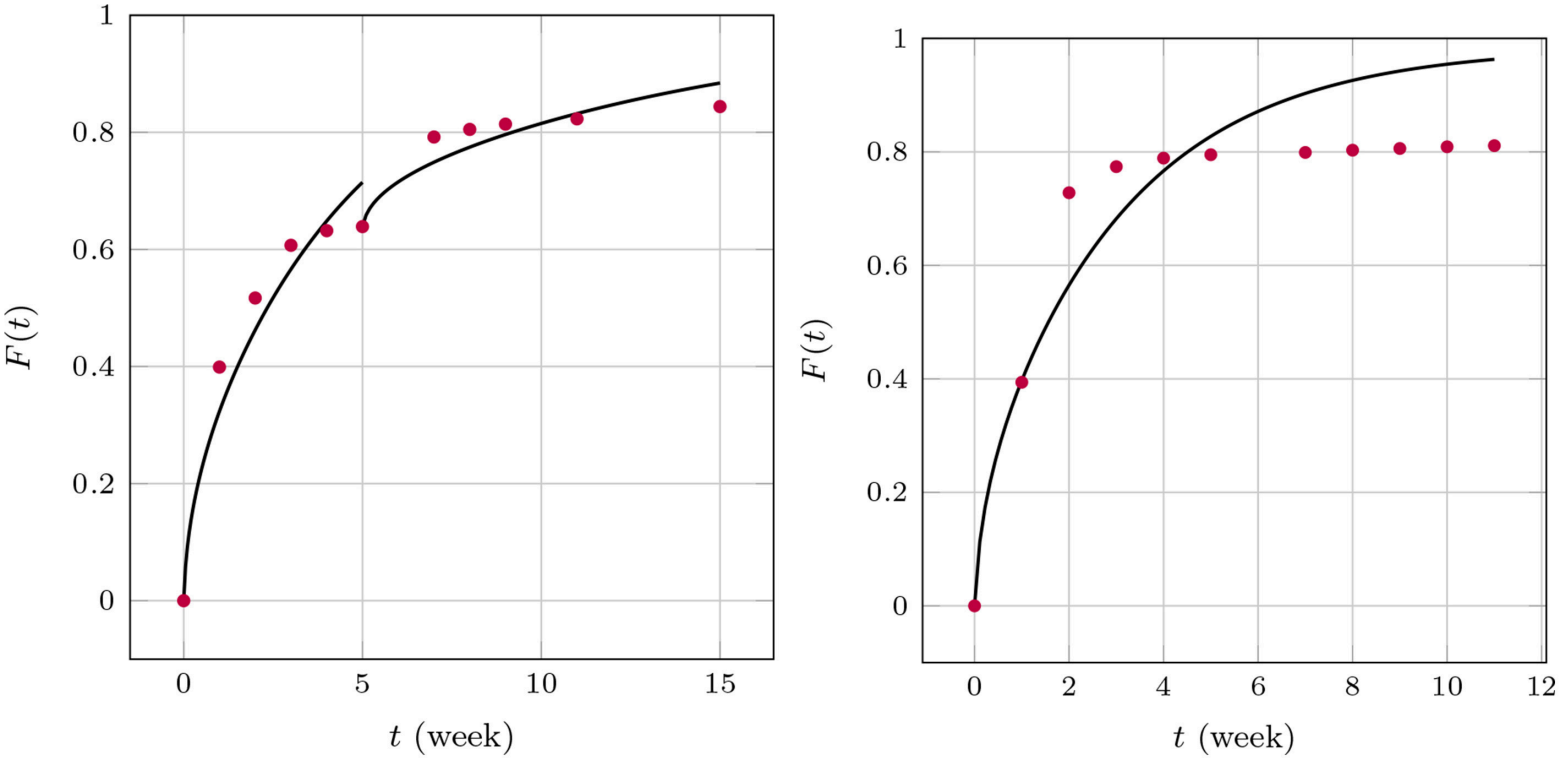
Predicted fractional release based on constant diffusion coefficients for GM **(Left)** and BP **(Right)**.

The apparent time-dependence of the diffusion coefficients shown in Figures 7, 9 has already been critically examined and interpreted as an artifact due to somewhat artificial realization of the boundary condition at the interface between the matrix and solution. The latter was based on a discontinuous fit of the observed values for *F*(*t*). It was mentioned that continuous boundary conditions cannot be captured within the framework of an analytical solution, such as the one shown in Equation (19) and numerical methods have to be employed. An FE study^2^ of the one-dimensional problem has therefore been performed with the objective to describe the temporal development of *F*(*t*) by means of a single value for the diffusion parameter *D*_m_. To this end, the discrete data *F*(*t*_*i*_) were interpolated linearly. The diffusion Equation (14) was solved repeatedly by changing the diffusion coefficient in combination with a Gauss mean square target function for the predicted and measured release data. The result is shown in Figure 10.

We found for GM that $D_{\text{m}}=1.47\times 10^{-9}\frac{{mm}^{2}}{s}$ and $D_{\text{m}}=0.66\times 10^{-9}\frac{{mm}^{2}}{s}$ for the first 5 weeks and after that, respectively, and $D_{\text{m}}=2.22\times 10^{-9}\frac{{mm}^{2}}{s}$ for BP. The least mean square error is around 2.5%, 1.3%, and 3.6% and therefore quite high. This is also indirectly visible in the plots when the predictions (solid lines) are compared to the measurements (red dots). Similarly as in the case of the averaged time dependent diffusion coefficients shown in Figure 7 the value of *D*_m_ decreases after the first 5 weeks when the diffusion is more impeded. However, the absolute numbers are now smaller.

**Figure 11:**
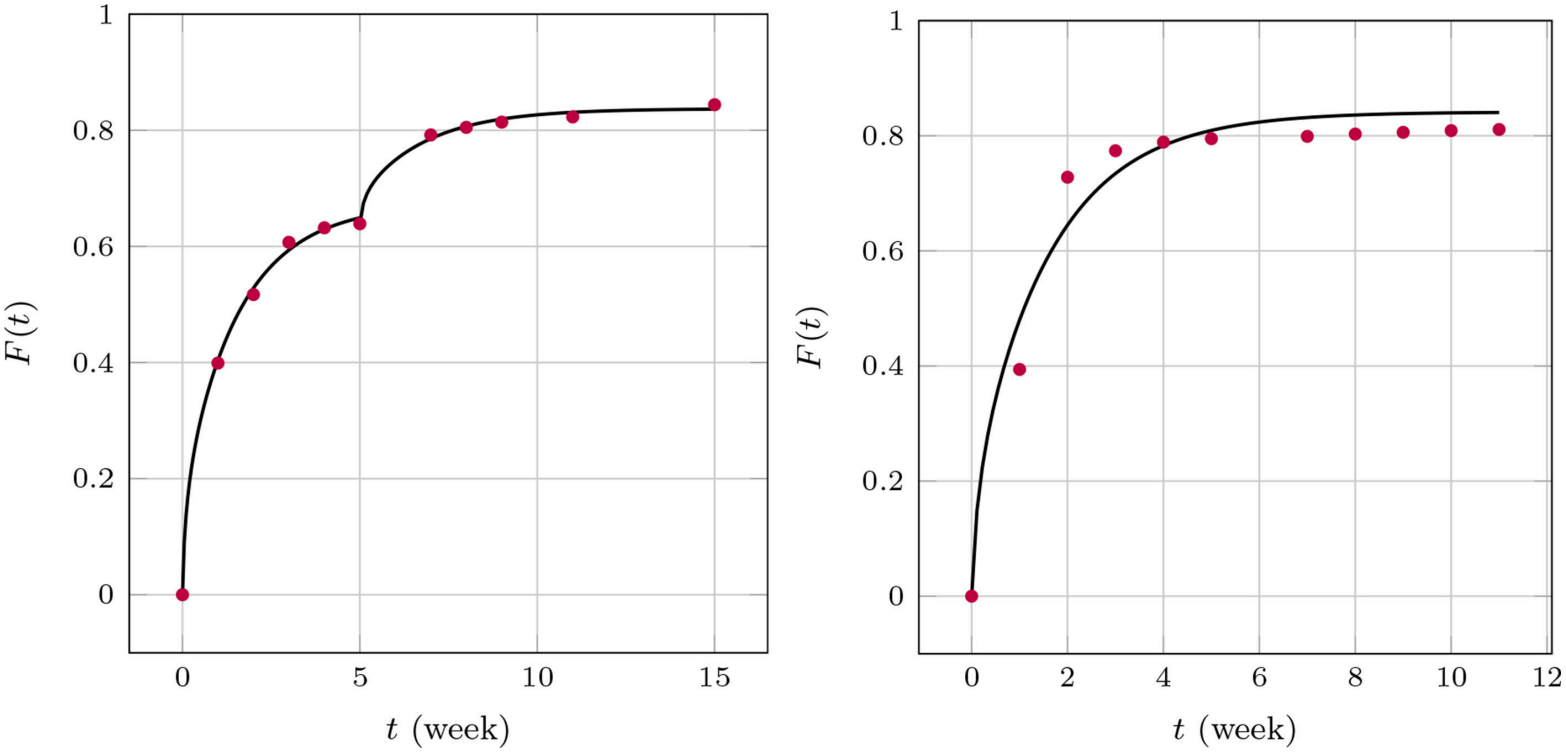
Predicted fractional release based on effective diffusion coefficients (Temkin model) for GM **(Left)** and BP **(Right)**.

We now turn to the solution of the diffusion equation when using the effective concentration dependent diffusion coefficient according to Temkin, Equation (7). After using the same FE and optimization techniques as before we obtained the results shown in Figure 11. We found for GM $D_{\text{m}}=3.91\times 10^{-9}\frac{{mm}^{2}}{s},A=2.66\times 10^{-3}\frac{\text{mg}}{\text{ml}}$ and $D_{\text{m}}=3.69\times 10^{-9}\frac{{mm}^{2}}{s},A=3.97\times 10^{-3}\frac{\text{mg}}{\text{ml}}$, respectively, and for BP $D_{\text{m}}=4.15\times 10^{-9}\frac{{mm}^{2}}{s},A=1.03\times 10^{-3}\frac{\text{mg}}{\text{ml}}$. Note that during the two stages of the GM release the *A* value changes. This is reasonable to assume because the absorption properties will change when the matrix structure alters. In fact absorption is increasing, as one could surmized by looking at the concentration data, which saturate earlier than anticipated. A decreasing value of *A* is in agreement with this. It is also interesting to note that the diffusion coefficient stays almost at the same level. Also, the agreement between predictions and measured data is now much better. Indeed, the least mean square errors of the predicted and the observed release values are 0.4, 0.2, 1.4 percent, respectively. The *D*_m_ of BP is also greater than those for GM, which corresponds to the smaller size of the drug molecule. Its absolute value is in agreement with the *D*_m_(*t*)-values at later stages shown Figure 9.

**Figure 12:**
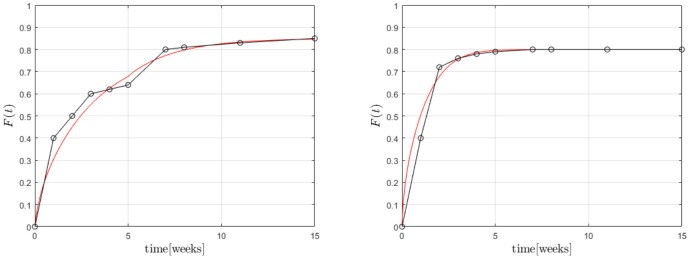
Predicted fractional release based on the Robin boundary condition (8) for GM **(Left)** and BP **(Right)**.

Finally Equations (3), (4) were solved (with $\dot{d}=0$) within the cylindrical region in combination with the boundary condition (8) based on an FV technique. The results are shown in Figure 12.

A first analysis predicted diffusion coefficients for GM of $D_{\text{m}}=2.33\times 10^{-9}\frac{{mm}^{2}}{s}$ for the whole time span. However, the coefficient α varied between $0.04\frac{ml}{\text{mg}}$, for the first five and $0.02\frac{ml}{\text{mg}}$ for the remaining weeks, respectively, while $c^{*}=0.425\frac{mg}{\text{ml}}$. This seems reasonable because the transfer is impeded after 5 weeks. α plays a similar role as *A* in the case of Temkin's concentration dependent diffusion coefficient. They are inverse to each other, so to speak. A full scale optimization analysis is currently underway, which might give a result similar to the one found for GM during the FE-calculations. In other words the *D*_m_ might prove to be almost equal during the two time zones, while the values of α are not. For BP we found $D_{\text{m}}=4.27\times 10^{-9}\frac{{mm}^{2}}{s}$, α = 0.1, $c^{*}=0.4\frac{mg}{\text{ml}}$. This is of the same magnitude as in the case of the FE-investigations.

## 4. Conclusions and Outlook

Marine structures can play a vital role in the treatment of a wide range of human diseases including non-curable one by providing hydrothermally converted coralline HAp for drug loading and release within a polymer-based matrix. In drug delivery, they have demonstrated a potential for a controlled release of clinically active agents. Their combination with degradable polymers in terms of a drug containment facility supplying the drug in a controlled manner to the polymer and then finally to the body fluid has widened their application in orthopedics as implant coatings for the prevention of biofilm.

In this paper experimental results have been presented showing the release behavior without and with such HAp containments. Moreover, the establishment of a quantitative drug release kinetics model can help to speed up the controlled drug release systems manufacturing. Knowing and quantitatively describing the complexity of mechanisms will lead to mastering the release from these devices.

For this reason, a collaborative effort between materials scientists and continuum physicists has been made for the development of a physically sound and closed-form release model, presently only for the case without HAp containment. The experimental results presented in this paper have been careful considered and related to the theoretical aspects in this model incorporating diffusion and degradation of the polymer matrix. Quantitative results for time-dependent diffusion coefficients in a degrading polymer matrix were presented based on this closed-form 1D diffusion model for a thin film curled up to form a thin-walled cylinder. The non-physical nature of the time-dependence was discussed and average values for the diffusion coefficients were compared to former literature data. In addition first numerical investigations, based on FE and FV methods were presented, which confirmed the average values of the time-dependent diffusion coefficients from the analytical model. The presented diffusion coefficients can be considered as geometry independent and they are ready for predicting the release kinetics in other geometries but thin film, for instance, fine structures made from 3D polymer printing. These are in preparation by the authors.

In the future the following remains to be done: First, the presented cylindrical solution model should be evaluated for a continuous input of drug concentrations at the cylinder wall over time from a deteriorating PA matrix as well as HAp containment. More precisely: This will require even more detailed numerical analyses, based on finite element, finite volume, or finite difference methods. The development of such tools is currently underway and will result in time-independent diffusion coefficients covering the time span until massive degradation of the polymer matrix and for times after. It can be expected that the (time-dependent) diffusion constants obtained in this work will serve as good starting values for the iteration procedure involved in the numerical approach. Ideally, a micro-model leading to a constitutive equation for a supply term of drug to the polymer matrix should be developed and then be included in an extended diffusion equation. This relation will have be solved numerically with suitable initial-boundary value data. This would allow to study the effect of a HAp containment supplying drug to the polymer matrix. In a third step this constitutive relation must be extended to account also for the deterioration of the HAp container requiring further numerical analysis. The general idea is to separate the various physical mechanisms and to obtain physically meaningful, geometry independent parameters by linking such models to experimental observations.

## Data Availability

All datasets generated for this study are included in the manuscript and/or the supplementary files.

## Author Contributions

IM and BB-N: experiments; WM and EV: model and theory development; AM: finite volume analysis; BA and WR: finite element analysis.

### Conflict of Interest Statement

The authors declare that the research was conducted in the absence of any commercial or financial relationships that could be construed as a potential conflict of interest.

## Footnotes

^1^Not to be confused with *D*_eff_ from Equation (7).

^2^The technical details of that study as well as the FV analysis mentioned further below will be published elsewhere.
